# Efficacy of pump-controlled selective antegrade cerebral perfusion in total arch replacement: A propensity-matched analysis

**DOI:** 10.3389/fsurg.2022.918461

**Published:** 2022-08-18

**Authors:** Yu Liu, Hui Jiang, Bin Wang, Zhonglu Yang, Lin Xia, Huishan Wang

**Affiliations:** Department of Cardiovascular Surgery, General Hospital of Northern Theater Command, Shenyang, China

**Keywords:** aortic dissection, selective antegrade cerebral perfusion, pump-controlled perfusion, upper hemisternotomy approach, total arch replacement

## Abstract

**Background:**

Pump-controlled selective antegrade cerebral perfusion (PC-SACP) in total arch replacement (TAR) can regulate cerebral flow accurately, which might be beneficial for cerebral protection. However, the safety of PC-SACP for TAR combined with frozen elephant trunk implantation (FET) in patients with acute Type A dissections (ATAAD) is ambiguous.

**Methods:**

A total of 192 patients with ATAAD underwent TAR at our institution from October 2019 to July 2021. The patients were divided into two groups based on PC-SACP used: PC group (SACP carried out by using a separate pump, *n* = 35) and Control group (SACP carried out as a traditional method, *n* = 157). Patients under PC-SACP were propensity-score matched to patients without PC-SACP, resulting in 35 pairs of patients.

**Results:**

Preoperative characteristics, including age, gender, weight, and preoperative creatinine level, were similar between the two groups. Cardiopulmonary bypass time, cross-clamp time, circulatory arrest time, and minimum nasopharyngeal temperature did not differ between the two groups. However, SACP time (54 versus 40, *P* = 0.001) in the PC group was significantly longer than that in the Control group. The incidence of temporary neurologic dysfunction (5.7% versus 8.6, *P* = 0.643) showed a no significantly lower trend in the PC group compared with the Control group. Other clinical outcomes showed no significant intergroup differences.

**Conclusions:**

PC-SACP in TAR is safe and feasible and might be beneficial for avoiding brain injury caused by “luxury” perfusion.

## Introduction

Neurologic protection during aortic arch surgery is a challenging strategy for improving clinical outcomes in patients with acute Type A aortic dissection (ATAAD) (1). Deep hypothermic circulatory arrest (DHCA)–induced electrocerebral inactivity has been thought to ensure optimal neuroprotection, which reached the minimum cerebral metabolic demand threshold (2). Moreover, the application of selective antegrade cerebral perfusion (SACP) has shifted the strategy from DHCA to moderate hypothermic circulatory arrest (MoHCA) or mild hypothermic circulatory arrest (MiHCA) (3, 4).

An earlier study demonstrated that bilateral SACP (BSACP) could maintain adequate cerebral blood supply at moderate hypothermia without an ischemic brain injury (5). The coupling of cerebral flow and metabolism is important for cerebral protection, and greater flow might increase the risk of cerebral edema and embolic phenomena (6). Although the suggested cerebral flow of selective cerebral perfusion is 6–10 ml/kg/min at 20–28 °C with a wide range of cerebral flow and temperature (7), our earlier study showed that 5 ml/kg/min of cerebral flow was sufficient for cerebral protection (8, 9), implying that excessive perfusion pressure and flow are unnecessary and should be avoided (6).

The application of SACP in the total arch replacement (TAR) surgery has been routinely carried out by using one main arterial line bifurcated for an SACP line and a systemic perfusion line via a Y connector (10). The flow of SACP and systemic perfusion depends on the resistance in cerebral arteries and systemic arteries, respectively. When the flow of the main arterial line is determined, occlusion of the systemic perfusion line can dramatically increase the flow of SACP, which may cause excessive SACP flow and pressure. This “luxury” perfusion might lead to brain injury. To solve this problem, a pump-controlled SACP (PC-SACP) has been implemented to avoid the excessive SACP that can occur during TAR surgery.

## Materials and methods

### Patients

Data were collected retrospectively from patients undergoing TAR for ATAAD. Patients with preoperative neurologic complications (cerebral infarction or cerebral hemorrhage), malperfusion syndrome, and concomitant operations (e.g., coronary heart disease, mitral valve disease, and congenital heart disease) were excluded. A total of 192 patients underwent TAR by the same surgery group, including surgeons, anesthetist, perfusionist, cardiologist, and nurses, at our institution between October 2019 and July 2021. The patients were divided into two groups: PC group (*n* = 35, PC-SACP was carried out) and Control group (*n* = 157, SACP carried out by a routine method) (Table 1). The study was approved by the Ethics Committee of General Hospital of Northern Theater Command, Shenyang, China. All patients provided their informed consent. The diagnosis of ATAAD was based on the patients’ clinical history and computed tomography angiography.

**Table 1:** Perioperative characteristics of patients.

Variable	Raw data			Matched data			
	PC group (*n* = 35)	Control group (*n* = 157)	*P* value	PC group (*n* = 35)	Control group (*n* = 35)	*P* value	*SMD*
**Preoperative characteristics**							
Age (years)	52.0 (44.0, 59.0)	54.0 (46.0, 60.0)	0.730	52.0 (44.0, 59.0)	52.0 (44, 59)	0.431	0.02
Male (%)	27 (77.1)	117 (74.5)	0.746	27 (77.1)	27 (77.1)	1.000	<0.001
Weight (kg)	74.0 (66.5, 88.0)	78.0 (65.0, 88	0.274	74.0 (65.0, 88.0)	75.0 (70.0, 85.0)	0.549	0.125
LVEF (%)	58.0 (58.0, 60)	58.0 (57.0, 59.5)	0.087	58.0 (58.0, 60.0)	58.0 (57.0, 59.0)	0.497	0.125
Smoking (%)	22 (62.9)	81 (51.6)	0.227	22 (62.9)	20 (57.1)	0.626	0.117
Diabetes (%)	2 (5.7)	11 (7.0)	0.783	2 (5.7)	3 (8.6)	0.643	0.111
Hypertension (%)	25 (71.4)	105 (66.9)	0.063	25 (71.4)	26 (74.3)	0.788	0.064
Pre-Cr (µmol/L)	71.0 (61, 81.0)	59 (46.7, 72.0)	0.001	71.0 (61.0, 81.0)	75.0 (70.0, 85.0)	0.137	0.008
**Intraoperative characteristics**							
CPB time (min)	160 (144, 191)	158 (140, 187.5)	0.307	160 (144, 191)	185 (156, 202)	0.987	0.08
Cross-clamp time (min)	101 (85, 128)	93 (78.5, 107)	0.043	101 (85, 128)	107 (95, 137)	0.787	0.003
Circulatory arrest (min)	10 (8, 13)	11 (5, 15)	0.838	10 (8, 13)	11 (8, 16)	0.354	0.458
BSACP (min)	53 (41, 58)	28 (23, 34)	0.000	53 (41, 58)	40 (31, 42)	0.001	1.057
Min nasopharyngeal T (°C)	30 (27, 32)	29 (28, 30)	0.003	30 (27, 32)	30 (29, 32)	0.644	0.109
Operative outcomes of patients							
Ventilation time (h)	19 (16, 39)	22 (18, 65.5)	0.029	19 (16, 39)	20 (17, 40)	0.701	
ICU stay (h)	43 (32, 87)	44 (24.5, 90)	0.775	43 (32, 87)	44 (35, 62)	0.614	
First 24-h chest tube drainage (ml)	260 (170, 650)	200 (150, 310)	0.441	260 (170, 650)	350 (200, 560)	0.993	
Perioperative blood transfusion	25 (71.4)	101 (64.3)	0.424	25 (71.4)	24 (68.6)	0.794	
Reoperation for bleeding (%)	1 (2.9)	4 (2.5)	0.917	1 (2.9)	1(2.9)	1.000	
Reventilation (%)	1 (2.9)	1 (0.6)	0.242	1 (2.9)	1 (2.9)	1.000	
TND (%)	2 (5.7)	22 (14)	0.179	2 (5.7)	3 (8.6)	0.643	
PND (%)	1 (2.9)	6 (3.8)	0.783	1 (2.9)	2 (5.7)	0.555	
Acute renal failure (%)	2 (5.7)	17 (10.8)	0.360	1 ()	3 (5.7)	1.000	
Paraplegia (%)	1 (2.9)	2 (1.3)	0.495	1 (2.9)	1(2.9)	0.314	
Postoperative length of stay (d)	14.0 (10.0, 19.0)	15.0 (11.0–19.0)	0.491	14 (10, 19)	16 (11, 21)	0.333	
In-hospital death (%)	3 (8.6)	12 (7.6)	0.853	3 (8.6)	2 (5.4)	0.643	

### Surgical procedure

**Figure 1:**
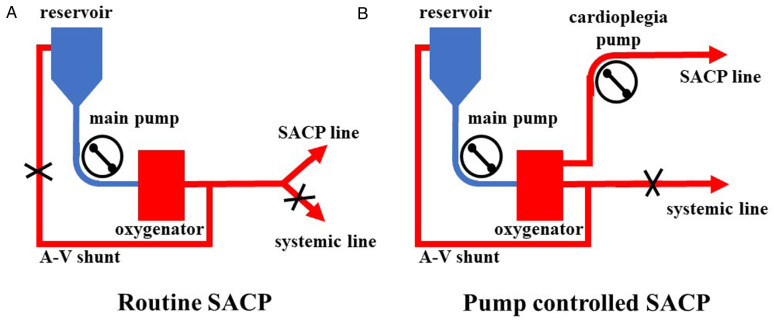
Application of pump-controlled SACP. (**A**) Routine SACP was carried out by clamping a systemic line and reducing flow of the main pump to 5 ml/kg·min. (**B**) Pump-controlled SACP was carried out by clamping the systemic line, opening A–V shunt, maintaining flow of the main pump at 2–3 L/min, and modulating cardioplegia pump to 5 ml/kg·min. SACP: selective antegrade cerebral perfusion.

**Figure 2:**
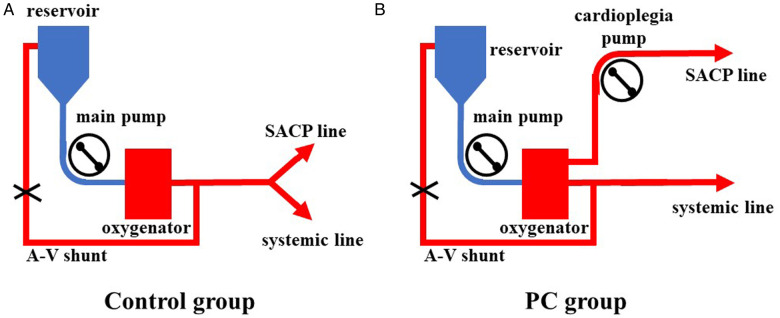
The application of LBP combined with SACP. (**A**) LBP and SACP were carried out by using the main pump together in the Control group. The cerebral flow was unclear. (**B**) LBP and SACP were carried out by using the main pump and the cardioplegia pump, respectively. LBP, lower body perfusion; SACP, selective antegrade cerebral perfuison; LBP, lower body perfusion; SACP, selective antegrade cerebral perfusion.

All surgeries were carried out via a single upper hemisternotomy approach, and near-infrared spectroscopy monitoring was used for cerebral protection during surgery, as in our earlier studies (8, 9). The cannulation strategy in the two groups was to select the innominate artery as the first artery perfusion cannula and the right atrial cannulation as the venous drainage cannula. The right-angle artery cannulation was selected for artery perfusion with the direction of blood flow to the heart. The right subclavian artery and the right or left common carotid artery were used as alternative cannulation sites. The cardioplegia strategy was aortic root or coronary orifices after aortotomy antegrade delivery. The right superior pulmonary vein was cannulated for left ventricular vent. Cardiopulmonary bypass (CPB) was carried out after cannulation, and MoHCA was induced at the time of aortic root procedures. The cerebral perfusion strategy was based on BSACP, which was carried out by using arterial cannulation, and a 15Fr femoral arterial cannula was placed into the left/right common carotid artery after the brachiocephalic arteries were cross-clamped. The blood flow control of SACP depended on the group. In one group, routine SACP was perfused through the main arterial pump as in previous studies (10, 11) with a systemic line clamped (Figure 1A). In the other group, PC-SACP was perfused by the cardioplegia pump (8, 12) with the systemic line clamped and A–V shunt opened (Figure 1B). The flow of SACP was approximately maintained at 5 ml/kg·min, which was modulated on the basis of near infrared spectroscopy (NIRS) monitoring. Then, the frozen elephant trunk (FET) with and without lower body perfusion (LBP) was carried out as in earlier reports (8, 13). Briefly, a stent graft (MicroPort Medical Co., Ltd., Shanghai, China) was mainly placed into the distal aorta after the origin of the left subclavian artery and the left carotid artery transected. Then, through a four-branch prosthetic graft (VASCUTEK Ltd., a Terumo Co., Inchinnan, Scotland), an endotracheal cannula (Teleflex Medical Ltd., Wayne, PA, USA) with an inside diameter of 5.5 mm was placed into the distal artery for delivering oxygenated blood to the lower body and preventing the backflow as LBP with 25 ml/kg·min of flow (8). Moreover, LBP was switched to the four-branch prosthetic graft after the stent graft was attached to the four-branch prosthetic graft. The sequence of anastomosis to the prosthetic graft was carried out from the left common carotid artery, proximal aortic stump, innominate artery, and the left subclavian artery in succession. Special attention was paid to carrying out cerebral perfusion by using the cardioplegia pump until the innominate artery was anastomosed in the PC group (Figure 2B). The cerebral perfusion and LBP were carried out by using the main pump together in the Control group (Figure 2A). After anastomosis to the left common carotid artery, CPB gradually returned, and rewarming started. Temporary pacing wire and a drainage tube were installed before sternal closure.

### Definitions for complications

For the purposes of this study, temporary neurologic dysfunction (TND) was defined as the presence of reversible postoperative motor deficit, confusion, or transient delirium with complete resolution of symptoms before discharge from the hospital. Permanent neurologic deficit (PND) was defined as the presence of either new stroke or coma with permanent neurological dysfunction confirmed by means of computed tomography of the brain. Postoperative renal dysfunction was defined as a creatinine level >230 µmol/L (twice the normal value). Perioperative blood transfusion was defined as intraoperative and postoperative transfusion of red blood cells, fresh frozen plasma, and platelets.

### Statistical analysis

Perioperative data were collected prospectively. Analyses were performed with SPSS version 22.0 software (SPSS, Inc., Chicago, IL). Normally distributed data were presented as group means ± SEM or SD, and non-normally distributed data were presented as the median and interquartile ranges. Student's *t*-test and Mann–Whitney *U* test were used to compare continuous variables. Categorical variables were analyzed by using the *χ*^2^ test or Fisher's exact probability test (if necessary). Differences with *P *< 0.05 were considered statistically significant.

Propensity score (PS) matching was conducted between the two groups to simulate randomization in this observational study. PS was estimated by using the logistic model and matched between the two groups within a caliper of 0.2 PS standard deviations. The covariates were based on eleven clinical variables, namely, gender, age, height, left ventricular ejection fraction (LVEF), preoperative creatinine level, and the history of hypertension, diabetes, stroke, CPB time, cross-clamp time, and minimum nasopharyngeal temperature. Then, standardized mean difference (SMD) was carried out for assessing the balance between the groups, and SMD < 0.25 was considered as “balance satisfied”. The Wilcoxon signed rank test was used to compare PS-matched pair variables.

## Results

### Baseline characteristics and propensity score matching

After PS matching, 70 patients (35 pairs) remained. The raw and matched data of preoperative and intraoperative characteristics are listed in Table 1. We did not observe any significant differences in preoperative and intraoperative characteristics between the two matched groups. Moreover, all time characteristics were found balanced by SMD.

### Intra- and postoperative characteristics

We did not find any differences in CPB time, cross-clamp time, CA time, and minimum nasopharyngeal temperature between the two groups. However, BSACP time (54 min versus 40 min, *P* = 0.001) in the PC group was significantly longer than that in the Control group (Table 1).

Three patients (8.6%) died in the PC group, and two patients (5.4%) died in the Control group. In the PC group, one patient died of multiple organ failure, and two patients died of sudden hemodynamic changes that were thought to be due to aortic rupture. One patient died of postoperative massive cerebral infarction and one patient died of multiple organ failure in the Control group. We did not observe any differences in ventilation time, ICU stay, postoperative in-hospital stay, and the incidence of acute renal failure and paraplegia between the two groups. The incidence of TND showed a lower trend in the PC group compared with the Control group (5.7% versus 8.6%), but without reaching statistical significance (*P* = 0.643), as well as the incidence of PND. Other postoperative characteristics, including chest tube drainage, the incidence of perioperative blood transfusion, reoperation for bleeding, and reventilation, did not show any differences between the two groups (Table 1).

## Discussion

Neurologic injury is a potentially devastating complication of ATAAD, and cerebral protection is vital during TAR surgery. SACP has been demonstrated as the best method of cerebral protection during TAR surgery (14–16). Different strategies of SACP affecting the outcomes include flow rates of SACP, unilateral or bilateral application, duration of DHCA, degree of hypothermia, and blood gas strategy (1, 6, 14, 17).

An intact circle of Willis is believed to be the base of unilateral SACP. However, incompleteness of the circle of Willis has been reported in up to 40% of patients (18). Earlier studies have demonstrated that BSACP offers better cerebral protection at a higher temperature (19–21). Therefore, BSACP was carried out in both groups to maintain the continuous cerebral perfusion through the innominate artery and the left common carotid artery, except when the left common carotid artery was anastomosed to the prosthetic graft. In the present study, we did not find any significant differences between the two groups, except for SACP time (53 min versus 40 min, *P *= 0.001). Because SACP time in the PC group was calculated from CA to the innominate artery anastomosed and the flow was controlled by using a cardioplegia pump throughout the process of SACP, the increased time was spent on the left common carotid and proximal aortic stump anastomosed. The routine SACP used a Y connector to separate the main arterial line to SACP and the systemic perfusion line. The flow between the SACP and the systemic perfusion line depended on the resistance of the two lines. In the Control group, SACP time was only during CA, and BSACP was initially controlled by using the main pump separately after the brachiocephalic arteries were cross-clamped. Then, the flow through the innominate artery was influenced by LBP when CA recovered, and the flow of LBP was controlled by using the main pump as well. So, the cerebral flow was unclear, leading to the possibility of “luxury” perfusion.

**Figure 3:**
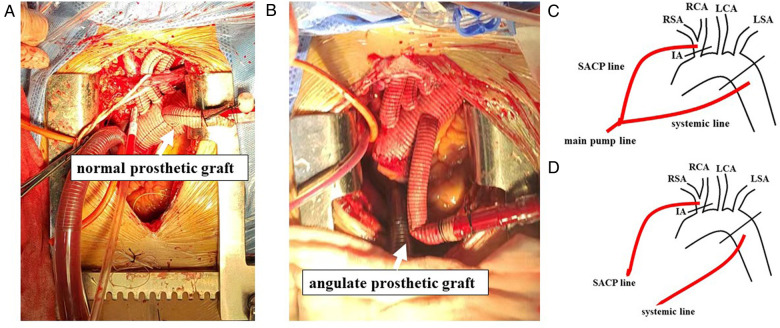
Angulated prosthetic graft and the advance of pump-controlled SACP. (**A**) A normal prosthetic graft for systemic (lower body) perfusion. (**B**) An angulated prosthetic graft for systemic (lower body) perfusion. (**C**) The main pump line split to the SACP line and the systemic line in the Control group. With the constant flow of the main pump, the decreased systemic flow caused by the angulated prosthetic graft increased SACP flow. (**D**) The main pump controlled the flow of the systemic line, while the cardioplegia pump controlled the flow of the SACP line. The angulated prosthetic graft influenced only the flow of the systemic line. The flow of the SACP line was always modulated accurately. SACP, selective antegrade cerebral perfusion; IA, innominate artery; RSA, right subclavian artery; RCA, right common carotid artery; LCA, left common carotid artery; LSA, left subclavian artery.

It is easy for a prosthetic graft to be out of shape. Thus, during surgery, some mistakes by surgeons caused the prosthetic graft to angulate, which increased the resistance of the systemic perfusion line and decreased the flow dramatically (Figure 3). Then, the pressure and flow of the SACP line to the innominate artery increased dramatically, which might have caused “luxury” perfusion to the brain and led to TND. This situation did not occur in the PC group because the cerebral flow was always controlled by using a separate pump. Although the prosthetic graft angulation might occur in the PC group, only the flow of LBP would decrease dramatically. The decreasing trend in the incidence of TND (5.7 versus 8.6, *P* = 0.643) might be caused by the effect of PC-SACP, which might be beneficial for cerebral protection, while also preventing “luxury” perfusion of the brain.

## Limitations

This study had some limitations. First, this was a single-center retrospective study, although propensity score analysis was used to simulate randomization. Second, the small sample size, especially in the PC group, might have caused confounder bias. Third, the perioperative characteristics were insufficient, which might have influenced the evaluation of clinical outcomes. Thus, a prospective larger-sample study is necessary.

## Conclusions

In this study, we found that PC-SACP in TAR was safe and feasible. This approach may be beneficial for avoiding neuroinjury caused by “luxury” perfusion.

## Data Availability

The raw data supporting the conclusions of this article will be made available by the authors without undue reservation.
